# COVID-19 vaccine acceptance and hesitancy in patients with Parkinson's disease

**DOI:** 10.3389/fpubh.2022.977940

**Published:** 2022-10-11

**Authors:** Yifan Zhou, Zhengyu Lin, Xiaonan Wan, Jun Liu, Jianqing Ding, ChenCheng Zhang, Kang Ren, Dianyou Li, Yiwen Wu

**Affiliations:** ^1^Department of Neurology and Institute of Neurology, Ruijin Hospital Affiliated to Shanghai Jiaotong University School of Medicine, Shanghai, China; ^2^Department of Neurosurgery, Center for Functional Neurosurgery, Ruijin Hospital Affiliated to Shanghai Jiaotong University School of Medicine, Shanghai, China; ^3^Gyenno Science Co., Ltd., Shenzhen, China

**Keywords:** COVID-19, vaccination, vaccine hesitancy, Parkinson's disease, SARS-CoV-2

## Abstract

**Background:**

As coronavirus disease 2019 (COVID-19) vaccination campaign underway, little is known about the vaccination coverage and the underlying barriers of the vaccination campaign in patients with Parkinson's disease (PD).

**Objective:**

To investigate the vaccination status and reasons for COVID-19 vaccine acceptance and hesitancy among PD patients.

**Methods:**

In concordance with the CHERRIES guideline, a web-based, single-center survey was promoted to patients with PD *via* an online platform from April 2022 and May 2022. Logistic regression models were used to identify factors related to COVID-19 vaccine hesitancy.

**Results:**

A total of 187 PD cases participated in this online survey (response rate of 23%). COVID-19 vaccination rate was 54.0%. Most participants had a fear of COVID-19 (77.5%) and trusted the efficacy (82.9%) and safety (66.8%) of COVID-19 vaccine. Trust in government (70.3%) and concerns about the impact of vaccine on their disease (67.4%) were the most common reasons for COVID-19 vaccine acceptance and hesitancy, respectively. COVID-19 vaccine hesitancy was independently associated with the history of flu vaccination (OR: 0.09, *p* < 0.05), trust in vaccine efficacy (OR: 0.15, *p* < 0.01), male gender (OR: 0.47, *p* < 0.05), disease duration of PD (OR: 1.08, *p* < 0.05), and geographic factor (living in Shanghai or not) (OR: 2.87, *p* < 0.01).

**Conclusions:**

The COVID-19 vaccination rate remained low in PD patients, however, most individuals understood benefits of vaccination. COVID-19 vaccine hesitancy was affected by multiple factors such as geographic factor, history of flu vaccination, disease duration and trust in efficacy of vaccine. These findings could help government and public health authorities to overcome the barrier to COVID-19 vaccination and improve vaccine roll-out in PD patients.

## Introduction

Coronavirus disease 2019 (COVID-19) is an infectious disease caused by the severe acute respiratory syndrome coronavirus 2 (SARS-CoV-2). As of June 2, 2022, there have been over 500 million confirmed cases of COVID-19 and 6.3 million deaths worldwide (https://covid19.who.int/). Emerging evidence has demonstrated that the elderly population is particularly vulnerable to SARS-CoV-2 infection (1). It has been shown that the risk for hospitalization, intensive care unit (ICU) and death due to COVID-19 continuously increases with age among people older than 40 years (2–4).

Parkinson's disease (PD) is now one of the fastest growing neurological diseases affecting ~6.1 million individuals worldwide in 2016 (5). The overwhelming majority of patients with PD are aged over 60 years. According to a recent nationwide report, the prevalence of PD in China was 1.37% in people older than 60 years, corresponding to a total estimated number of 3.6 million PD cases (6). The current COVID-19 pandemic has raised extensive concerns among neurologists, some of whom have warned that the world healthcare systems should be ready for the third wave of parkinsonism as influenza has long been considered as a potential driver in PD pathogenesis (7, 8). It has been suggested that influenza (e.g., the Spanish Flu) is associated with an increased risk of PD (9, 10). In addition, the COVID-19 mortality rate is found to be higher in PD patients as compared to the general elderly population (11, 12). Moreover, patients with PD are more likely to experience worsening motor and non-motor symptoms in the setting of SARS-CoV-2 infection (13). Consistent with clinical observations, basic research also provided intriguing insights into the association between COVID-19 and PD pathogenesis (14–16). The α-synuclein (α-syn) aggregation is the most critical driver in PD development. Recent vitro studies reported that SARS-CoV-2 protein can directly interact with α-syn and accelerate the formation of α-syn aggregation (14, 16). The short- and long-term impact of COVID-19 on PD was demonstrated in rodent models, in which the neuronal loss and microglia activation was more severe in PD mice infected and recovered from SARS-CoV-2 infection (15). In order to protect PD patients from COVID-19, the International Parkinson's and Movement Disorder Society (IPMDS) strongly recommends COVID-19 vaccination for PD patients unless they have a specific contraindication (17).

With COVID-19 vaccine roll-out underway, the vaccination coverage in China has reached 91.22% nationwide and 86.23% in people over 60 years of age. However, little is known about the COVID-19 vaccination status among patients with PD in China. Given the low flu vaccination willingness reported in PD patients, it is reasonable to assume that there may exist a barrier that prohibits patients from accepting and receiving the COVID-19 vaccine (18). In addition, several case studies has observed the occurrence of functional psychogenic-neurological disorders (FNDs) in healthy recipients and worsening motor symptoms in PD patients following COVID-19 vaccination, which may further create a negative impression on COVID-19 vaccine among the public (19, 20).

Widespread public acceptance and population coverage are foundations for the success of COVID-19 vaccination campaign. It is worth mentioning that though the Chinese government has encouraged the elderly to receive vaccination for a year, the vaccination coverage rate in people older than 60 years remain relatively low in Shanghai (21). The present study aimed to investigate the COVID-19 vaccination status, reasons for vaccine acceptance/hesitancy and factors related to vaccine hesitancy in PD patients. To explore whether PD patients in Shanghai was similarly at risk of vaccine hesitant compared to those living in other cities and the reasons underlying Shanghai's low vaccination willingness, participants was divided into patients in Shanghai and those in other cities. This study may help public health agencies develop strategies to improve vaccination coverage and protect patients' health.

## Methods

### Survey design and study participants

A cross-sectional, web-based online survey was conducted according to the CHERRIES guideline between April 25, 2022 and May 2, 2022. To guarantee honest feedback, a self-reported, anonymous questionnaire entitled “COVID-19 vaccines and Parkinson's disease” was developed and distributed *via* the domestic largest social platform (Wechat, Tencent Co., Ltd., Shenzhen, China). Briefly, the questionnaire was randomly promoted to 813 patients with regular follow-up at the Movement Disorder Clinic of Ruijin Hospital (Shanghai Jiao Tong University School of Medicine, Shanghai, China) and fulfilled the UK Brain Bank criteria. Patients were all informed the purpose of study and were asked to voluntarily answer the questionnaire at their convenience. Participants could review and change their answers before clicking the submit button. A total of 188 patients submitted the questionnaire by the end of study. One respondent was excluded due to the incompleteness of the submitted questionnaire. Therefore, the response rate was 23% (187/813). Each respondent was confirmed to be a unique individual by their IP addresses and telephone numbers. The study was approved by the institutional review board of Ruijin Hospital. Online informed consent was obtained from all participants.

### Data collection

The content of the questionnaire included: (1) socio-demographic and clinical data; (2) history of COVID-19; (3) history of COVID-19 vaccination and flu vaccination; (4) attitudes toward COVID-19 and COVID-19 vaccines; (5) reasons for COVID-19 vaccines acceptance and hesitancy. Sections of 2-5 were queried through multiple-choice. To assess the attitudes toward COVID-19 (**Table 2**), participants were asked the following questions: “Are you afraid if you/your family get SARS-CoV-2 infection?” and “Do you agree that asymptomatic COVID-19 individuals cannot infect others?” Correct attitude was defined as “a little or very” and “disagree”, respectively. Participants were further asked “Do you wear a mask/avoid taking public transport/ avoid going out/maintain social distance from people?”. Correct attitude was defined as “yes”. The following questions were presented to assess the attitudes toward COVID-19 vaccines: “Do you agree that COVID-19 vaccine is important for health?” and “Do you agree that COVID-19 vaccine is safe?” and the right attitude was defined as “agree”.

Since the nucleic acid amplification test is more readily accessible than antigen test in China, the diagnosis of COVID-19 was confirmed based on positive nucleic acid amplification test results. Additionally, sticking with the “dynamic zero” policy, residents in China are asked to take PCR test every 2–3 days. Therefore, a positive PCR result is available in most of the cases. In this study, symptomatic patients with negative laboratory results were not defined as SARS-CoV-2 infection. Patients who had already received or planned to receive COVID-19 vaccination were classified as the vaccine acceptance group. Patients who were reluctant or refused to receive the COVID-19 vaccination despite the availability of vaccination services were defined as vaccine hesitancy group. Their reasons for vaccine acceptance and hesitancy were shown in the **Tables 3**, **4**.

### Statistical analysis

Statistical analyses were performed on SPSS version 28.0 (SPSS, Chicago, IL, USA). The chi-square test and Fisher's exact test were used to compare categorical variables and the Student's *t*-test were used for continuous variables. Univariate logistic regression analyses were used to explore potential factors associated with vaccine hesitancy, in addition, variables with *p* < 0.05 were further entered into a multivariate analysis. A two-tailed *p* < 0.05 was defined as statistically significant.

## Results

### Study participants

Of the 187 participants, 46.0% (86/187) lived in Shanghai (SH-PD group) and 54.0% (101/187) lived in other cities (non-SH-PD group). Socio-demographic and clinical characteristics are listed in Table 1. The mean age was 64.2 years (SD 9.2) in total cohort with a gender ratio (male/female) of 1.79. Co-existing psychiatric issues were reported by five participants. Two participants claimed that they had concurrent depression and two had anxiety disorders. None of these cases was clinically confirmed in the study period. Compared with the non-SH-PD group, the SH-PD group was older in age (*p* < 0.0001) and had a higher education level (*p* < 0.01). No differences were found in gender ratio, PD duration, comorbidity, or cohabitation status between the two groups.

**Table 1:** Socio-demographic and clinical characteristics of patients with PD.

	**Total cohort (*n* = 187)**	**SH-PD (*n* = 86)**	**Non-SH-PD (*n* = 101)**	***P*** **-value**
**Age, years, mean (SD)**	64.2 (9.6)	67.3 (7.2)	61.5 (10.5)	**<0.0001**
**Gender ratio (M/F)**	1.79	1.96	1.65	0.58
**PD duration**, ***n*** **(%)**				
≤5 years	7 (3.7)	1 (1.2)	6 (5.9)	0.38
5–10 years	52 (27.8)	23 (26.7)	29 (28.7)	
10–20 years	111 (59.3)	53 (61.6)	58 (57.4)	
20–30 years	13 (7.0)	6 (7.0)	7 (6.9)	
≥30 years	4 (2.1)	3 (3.5)	1 (1.0)	
**Comorbidity**, ***n*** **(%)**				
No	80 (42.8)	35 (40.7)	45 (44.6)	0.60
Hypertension/diabetes	50 (26.7)	27 (31.4)	23 (22.8)	0.18
Neurological/psychiatric disorders	17 (9.1)	10 (11.6)	7 (6.9)	0.31
Orthopedic disorders	13 (7.0)	7 (8.1)	6 (5.9)	0.58
Others	45 (24.1)	24 (27.9)	21 (20.8)	0.26
Not acquired	2 (1.1)	1 (1.2)	1 (1.0)	>0.99
**Cohabitation status**, ***n*** **(%)**				
Alone	7 (3.7)	3 (3.5)	4 (4.0)	0.46
With family members	176 (94.1)	81 (94.2)	95 (94.1)	0.60
Nursing home	4 (2.1)	2 (2.3)	2 (2.0)	0.34
**Education attainment**, ***n*** **(%)**				
High school or lower	97 (51.9)	34 (39.5)	63 (62.3)	**<0.01**
College or higher	88 (47.1)	50 (58.1)	38 (37.6)	
Not acquired	2 (1.1)	2 (2.3)	0 (0)	

The bold value indicates the *p* value < 0.05.

### History of COVID-19

Among the participants, four patients (2.1%, 4/187) had a history of SARS-CoV-2 infection, including three in the SH-PD group (3.5%, 3/86) and one in the non-SH-PD group (1.0%, 1/101). In addition, three patients (1.6%, 3/187) in the SH-PD group reported that their family members were victims of COVID-19. No statistical difference in SARS-CoV-2 infection rate was detected between the SH-PD and non-SH-PD groups (Table 2).

**Table 2:** History and attitude of COVID-19 and COVID-19 vaccine.

	**Total cohort (*n* = 187)**	**SH-PD (*n* = 86)**	**Non-SH-PD (*n* = 101)**	***P*** **-value**
**A history of COVID-19**, ***n*** **(%)**	4 (2.1)	3 (3.5)	1 (1.0)	0.34
**Family member with a history of COVID-19**, ***n*** **(%)**	3 (1.6)	3 (3.5)	0 (0)	0.10
**Received flu vaccine in past 3 years**, ***n*** **(%)**	23 (12.3)	5 (5.8)	18 (17.8)	**0.01**
**Received COVID-19 vaccine**, ***n*** **(%)**	101 (54.0)	33 (38.4)	68 (67.3)	**< 0.001**
**People around received COVID-19 vaccine**, ***n*** **(%)**				
Yes	173 (92.5)	78 (90.7)	95 (94.1)	0.69
No	6 (3.2)	3 (3.5)	3 (3.0)	
Uncertain	8 (4.3)	5 (5.8)	3 (3.0)	
**Attitude toward COVID-19 and vaccine**				
1. **“Are you afraid if you get SARS-CoV-2 infection?”**, ***n*** (%)*				
No	42 (22.5)	7 (8.1)	35 (34.7)	**<0.001**
A little	109 (58.3)	59 (68.6)	50 (49.5)	
Very	36 (19.3)	20 (23.2)	16 (15.8)	
2. **“Are you afraid if your family get SARS-CoV-2 infection?”**, ***n*** (%)*				
No	33 (17.6)	6 (7.0)	27 (26.7)	**< 0.001**
A little	105 (56.1)	51 (59.3)	54 (53.5)	
Very	49 (26.2)	29 (33.7)	20 (19.8)	
3. **“Do you agree that COVID-19 vaccine is important for health?”**, ***n*** (%)*				
Agree	155 (82.9)	71 (82.6)	84 (83.2)	0.94
Disagree	3 (1.6)	1 (1.2)	2 (2.0)	
Ucertain	29 (15.5)	14 (16.3)	15 (14.9)	
4. **“Do you agree that COVID-19 vaccine is safe?”**, ***n*** (%)*				
Agree	125 (66.8)	51 (59.3)	74 (73.3)	0.12
Disagree	8 (4.3)	5 (5.8)	3 (3.0)	
Uncertain	54 (28.9)	30 (34.9)	24 (23.8)	
5. **“Do you agree that asymptomatic COVID-19 individuals cannot infect others?”**, ***n*** (%)*				
Agree	7 (3.7)	3 (3.5)	4 (4.0)	0.67
Disagree	144 (77.0)	64 (74.4)	80 (79.2)	
Uncertain	36 (19.3)	19 (22.1)	17 (16.8)	
6. **“Do you wear a mask when you go out?”**, ***n*** (%)*				
Yes	182 (97.3)	84 (97.7)	98 (97.0)	0.51
No	3 (1.6)	2 (2.3)	1 (1.0)	
Unclear	2 (1.1)	0 (0)	2 (2.0)	
7. **“Do you try to avoid taking public transport when you go out?”**, ***n*** (%)*				
Yes	170 (90.9)	79 (91.9)	91 (90.1)	>0.99
No	1 (0.5)	0 (0)	1 (1.0)	
Unclear	16 (8.6)	7 (8.1)	9 (8.9)	
8. **“Do you try to avoid going out?”**, ***n*** (%)*				
Yes	182 (97.3)	85 (98.8)	97 (96.0)	0.08
No	1 (0.5)	1 (1.2)	0 (0)	
Unclear	4 (2.1)	0 (0)	4 (4.0)	
9. **“Do you try to keep social distance from people?”**, ***n*** (%)*				
Yes	178 (95.2)	84 (97.7)	94 (93.1)	0.27
No	3 (1.6)	0 (0)	3 (3.0)	
Unclear	6 (3.2)	2 (2.3)	4 (4.0)	

*All “you” referred to the PD patient in the questionnaire.

The bold value indicates the *p* value < 0.05.

### History of COVID-19 vaccination and flu vaccination

A total of 101 participants (54.0%, 101/187) had received COVID-19 vaccination and 23 patients (12.3%, 23/187) had received flu vaccination in the past 3 years. Regarding the COVID-19 vaccination regimen, 46.0% (86/187) had not been vaccinated yet, 3.2% (6/187) had been vaccinated once, 19.8% (37/187) had been vaccinated twice and 31.0% (58/187) had received a booster shot.

**Figure 1:**
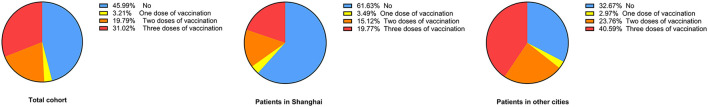
COVID-19 vaccination status in patients with PD. The COVID-19 vaccination status in patients with PD is shown with one dose of vaccination in yellow, two doses of vaccination in orange and three doses of vaccination in red. The percentage of patients without vaccination is presented in blue.

The COVID-19 vaccination rate and flu vaccination rate were markedly lower in the SH-PD group than that in the non-SH-PD group (38.4 vs. 67.3%, *p* < 0.001; 5.8 vs. 17.8%, *p* = 0.01, respectively). In addition, the percentage of patients who had received a booster COVID-19 vaccination was significantly lower in the SH-PD group (19.8 vs. 40.6%, *p* < 0.01) (Table 2; Figure 1).

### Attitude toward COVID-19 and COVID-19 vaccine

The majority of participants feared SARS-CoV-2 infection in themselves (77.5%, 145/187) and in their families (82.4%, 154/187). During the current COVID-19 epidemic, over 90% of patients took the following measures to minimize their risk of infection: wearing a mask (97.3%, 182/187), avoiding public transport (90.9%, 170/187), avoiding going out (97.3%, 182/187), and maintaining social distance from people (95.2%, 178/187). Most patients could understand the health benefits of COVID-19 vaccination (82.9%, 155/187) and trusted the safety of the COVID-19 vaccine (66.8%, 125/187).

The percentage of patients who had no fear of SARS-CoV-2 infection in themselves or in their families was markedly higher in the SH-PD group (*p* < 0.001 and *p* < 0.001, respectively). Compared to the non-SH-PD group, the SH-PD group was less likely to trust the safety of COVID-19 vaccines, although the difference did not reach statistical significance (59.3 vs. 73.3%, *p* = 0.12) (Table 2).

### Reasons for COVID-19 vaccine acceptance and hesitancy

Thirty-three SH-PD patients (38.4%, 33/86) and 68 non-SH-PD patients (67.3%, 68/101) were classified into the vaccine acceptance group. Fifty-three SH-PD participants (61.6%, 53/86) and 33 non-SH-PD participants (32.7%, 33/101) were classified into the vaccine hesitancy group. The COVID-19 vaccine acceptance rate was significantly higher in the non-SH-PD group (*p* < 0.001).

Reasons for COVID-19 vaccine acceptance and hesitancy are presented in Tables 3, 4. The most common reason for vaccine acceptance was trust in government (70.3%), followed by the intention to protect others (47.5%), trust in the safety of vaccine (38.6%), influence from others who had received the COVID-19 vaccination (30.7%), recommendations by specialists (25.7%), the intention to protect themselves (22.8%), and free of charge (15.8%). The most prevalent reason for COVID-19 vaccine hesitancy was concerns regarding the impact of vaccine on PD and/or other comorbidities, which was followed by suggestions from specialists (18.6%), inconvenience or difficulty in accessing the vaccination (15.1%), lack of trust in the safety of vaccine (11.6%) and old age (10.5%). The majority of patients with COVID-19 vaccine hesitancy stated that they would be willing to receive COVID-19 vaccination following recommendations by specialists (77.9%), the government (58.1%), and their family/friends (55.8%).

**Table 3:** Reasons for vaccine acceptance.

	**Total (*n* = 86)**	**SH-PD (*n* = 53)**	**Non-SH-PD (*n* = 33)**	***P*** **-value**
Trust in the government, *n* (%)	71 (70.3)	19 (57.6)	52 (76.5)	0.06
Willingness for protecting others from COVID-19, *n* (%)	48 (47.5)	13 (39.4)	35 (51.5)	0.29
Trust in the safety of vaccine, *n* (%)	39 (38.6)	9 (27.3)	30 (44.1)	0.13
Willingness of people around to be vaccinated (%)	31 (30.7)	9 (27.3)	22 (32.4)	0.65
Specialist recommandation, *n* (%)	26 (25.7)	5 (15.2)	21 (30.9)	0.14
Willingness for self-protection from COVID-19, *n* (%)	23 (22.8)	11 (33.3)	12 (17.6)	0.13
The vaccine is free of charge, *n* (%)	16 (15.8)	4 (12.1)	12 (17.6)	0.57

**Table 4:** Reasons for vaccine hesitancy.

	**Total (*n* = 101)**	**SH-PD (*n* = 33)**	**Non-SH-PD (*n* = 68)**	***P*** **-value**
**Concern about PD and/or comorbidity**, ***n*** **(%)**	58 (67.4)	38 (71.7)	20 (60.6)	0.35
**Suggestions from specialists**, ***n*** **(%)**	16 (18.6)	10 (18.9)	6 (18.2)	>0.99
**Inconvenient accessibility of vaccination (time/distance)**, ***n*** **(%)**	13 (15.1)	10 (18.9)	3 (9.1)	0.35
**Lack of trust in safety of vaccine**, ***n*** **(%)**	10 (11.6)	7 (13.2)	3 (9.1)	0.73
**Concern about old age**, ***n*** **(%)**	9 (10.5)	6 (11.3)	3 (9.1)	>0.99
**Suggestions from others other than specialists**, ***n*** **(%)**	8 (9.3)	4 (7.5)	4 (12.1)	0.48
**There is no risk of COVID-19**, ***n*** **(%)**	7 (8.1)	4 (7.5)	3 (9.1)	>0.99
**There is no serious consequence of COVID-19**, ***n*** **(%)**	2 (2.3)	1 (1.9)	1 (3.0)	>0.99
**Other reasons**, ***n*** **(%)**	6 (7.0)	4 (7.5)	2 (6.1)	>0.99
**Lack of trust in efficacy of vaccine**, ***n*** **(%)**	1 (1.2)	1 (1.9)	0 (0)	>0.99
**If the government recommends, are you willing to receive COVID-19 vaccine?**				
Yes	50 (58.1)	30 (56.6)	20 (60.6)	0.91
No	15 (17.4)	9 (17.0)	6 (18.2)	
Uncertain	21 (24.4)	14 (26.4)	7 (21.2)	
**If specialists recommend, are you willing to receive COVID-19 vaccine?** ***n*** **(%)**				
Yes	67 (77.9)	42 (79.2)	25 (75.8)	0.93
No	9 (10.5)	5 (9.4)	4 (12.1)	
Uncertain	10 (11.6)	6 (11.3)	4 (12.1)	
**If your family and friends recommend, are you willing to receive COVID-19 vaccine?** ***n*** **(%)**				
Yes	48 (55.8)	27 (50.9)	21 (63.6)	0.50
No	20 (23.3)	13 (24.5)	7 (21.2)	
Uncertain	18 (20.9)	13 (24.5)	5 (15.2)	

The types and prevalence of reasons for COVID-19 vaccine acceptance or hesitancy were similar between the SH-PD and non-SH-PD groups.

### Factors associated with COVID-19 vaccine hesitancy

In the univariate analysis, COVID-19 vaccine hesitancy was significantly associated with the history of flu vaccination (OR: 0.10, 95% CI: 0.023–0.443, *p* < 0.01), trust in the efficacy of vaccine (OR: 0.24, 95% CI: 0.097–0.617, *p* < 0.01), male gender (OR: 0.48, 95% CI: 0.256–0.903, *p* < 0.05), trust in the safety of vaccine (OR: 0.51, 95% CI: 0.267–0.961, *p* < 0.05), age (OR: 1.06, 95% CI: 1.021–1.097, *p* < 0.01), disease duration of PD (OR: 1.11, 95% CI: 1.044–1.171, *p* < 0.001), and grouping (SH-PD group) (OR: 3.72, 95% CI: 1.979–6.981, *p* < 0.001). The multivariable analyses indicated that vaccine hesitancy was independently associated with the history of flu vaccination (OR: 0.09, 95% CI: 0.015–0.581, *p* < 0.05), trust in vaccine's efficacy (OR: 0.15, 95% CI: 0.043–0.537, *p* < 0.01), male gender (OR: 0.47, 95% CI: 0.22–0.984, *p* < 0.05), disease duration (OR: 1.08, 95% CI: 1.014–1.157, *p* < 0.05) and grouping (SH-PD group) (OR: 2.87, 95% CI: 1.369–6.021, *p* < 0.01) (Table 5).

**Table 5:** Factors associated with COVID-19 vaccine hesitancy.

	**Univariable analysis**		**Multivariable analysis**	
	**OR (95%CI)**	***P*** **-value**	**OR (95%CI)**	***P*** **-value**
Received flu vaccination in the past 3 years	0.10 (0.023–0.443)	**<0.01**	0.093 (0.015–0.581)	**0.01**
Trust in safety of vaccine	0.51 (0.267–0.961)	**0.04**	1.086 (0.456–2.584)	0.85
Trust in efficacy of vaccine	0.24 (0.097–0.617)	**<0.01**	0.15 (0.043–0.537)	**<0.01**
Fear of family members being infected with SARS-CoV-2	1.50 (0.668–3.384)	0.33	-	-
Fear of SARS-CoV-2 infection	1.86 (0.88–3.918)	0.10	-	-
Comorbidity	1.27 (0.694–2.319)	0.44	-	-
PD disease duration	1.11 (1.044–1.171)	**<0.001**	1.08 (1.014–1.157)	**0.02**
Age	1.06 (1.021–1.097)	**<0.01**	1.03 (0.989–1.077)	0.15
Gender (male)	0.48 (0.256–0.903)	**0.02**	0.47 (0.22–0.984)	**0.04**
Geographic factor (living in Shanghai)	3.72 (1.979–6.981)	**<0.001**	2.87 (1.369–6.021)	**<0.01**
Education attainment	0.70 (0.385–1.284)	0.25	-	-

The bold value indicates the *p* value <0.05.

## Discussion

The elderly population, especially those with underlying diseases, presents a high case-mortality rate and poor prognosis in the setting of COVID-19 (2–4). COVID-19 vaccination has proven to be safe and effective in preventing infection and reducing the risks of illness, hospitalization and death. Based on data from real-life and clinical trials, most of the approved COVID-19 vaccines are highly effective (>70%) in people older than 60 years of age. A complete schedule of COVID-19 vaccination has been shown to result in a higher magnitude of neutralizing antibodies and effectiveness than a single vaccination dose. Regarding the safety of COVID-19 vaccines, mild to moderate self-limiting side-effects (e.g., fever) have been documented in the elderly. The incidence of side-effects seems to be lower in older recipients, and severe adverse events are very rare (22–24). To protect older adults from COVID-19, the Chinese government has been devoted to facilitating COVID-19 vaccination coverage for months. According to the National Health Commission (NHC) of China, as of March 17, 2022, the proportion of people who received one dose of COVID-19 vaccination among individuals aged 60–69, 70–79, and over 80 years, was 88.8, 86.1, and 58.8% respectively; the proportion of people with complete course of basic immunization was 86.6, 81.7, and 50.7%, respectively, and the proportion of people with a booster vaccination was 56.4, 48.4, and 19.7%, respectively (http://www.nhc.gov.cn/). Our study indicated that the vaccination coverage was even lower in PD patients as compared to the general elderly population, with only 54.0% of PD cases receiving ≥1 dose of COVID-19 vaccination and 30.0% receiving a booster shot. Although vaccine hesitancy was identified in nearly half of the PD patients, most patients believed that the COVID-19 vaccines available in China were effective and safe. Most patients feared being infected with by SARS-CoV-2. Therefore, they complied with several recommended physical measures to reduce the risk of COVID-19. These findings suggests that most PD patients were fully aware of the dangers of COVID-19 and the importance of COVID-19 vaccination, but were hesitant to be vaccinated.

The two most prevalent reasons for COVID-19 vaccine hesitancy were concerns about the impact of vaccine on their disease and suggestions from specialists. In fact, the Technical Guideline for Vaccination Against SARS-CoV-2, published by the NHC of China, recommends COVID-19 vaccination for people older than 60 years and for patients with chronic diseases. This guideline further states that people with uncontrolled epilepsy and other serious nervous system diseases (such as transverse myelitis, Guillain Barre syndrome, and demyelinating diseases) are not recommended to receive the COVID-19 vaccination. This contradiction notion is ambiguous and, to some extent, leaves patients and even specialists uncertain about whether people with PD or other neurological comorbidities (e.g., stroke) should be vaccinated. In addition, there is a lack of clinical trials assessing the safety and efficacy of COVID-19 vaccines in elderly people with extreme old age, frailty and comorbidity (25–27). Based on sparse case reports, COVID-19 vaccine recipients may experience neurological symptoms classified as functional neurological disorders (19). Moreover, several case studies have reported worsening motor symptoms in PD patients and movement disorders non-PD patients following COVID-19 vaccination (20, 26–29). On the one hand, these observations indicate that side-effects of COVID-19 vaccines are not fully documented and highlight the need for post-injection surveillance and long-term monitoring among vaccine recipients. On the other hand, these reports, if not adequately interpreted, may raise public concerns about the current COVID-19 vaccination campaign and reduce their willingness to be vaccinated.

Taking the COVID-19 vaccination is in essence a trade-off between the benefits of the vaccine and the risks of its side-effects. The current COVID-19 vaccines have proven to be safe for healthy older adults, however, there is a lack of clinical data that specifically evaluates the safety of vaccines on persons with PD. A recent study by Solda et al. recruited 34 PD patients and found that most adverse events of COVID-19 vaccines were mild and, compared with the control group, the incidence of adverse events was significantly lower in the PD group (30). Deteriorated PD symptoms and new-onset movement disorders (e.g., tremor) in non-parkinsonism patients following COVID-19 vaccination have been highlighted in several case reports (26–29). Notably, the neurological side-effects described in these cases were transient and completely resolved with appropriate intervention. However, the mechanisms underlying post-vaccination neurological complications remains unclear. No causal relationship between COVID-19 vaccination and worsening PD symptoms could be established based on current evidence. Whether these side-effects are induced by functional brain network dysfunctions or are elicited by systemic inflammatory responses remains unknown. Taken together, COVID-19 vaccines appear to be safe and tolerable for patients with PD. More clinical data are clearly needed to clarify the safety of COVID-19 vaccines among PD patients in future investigations. Given that the elderly people and PD patients are particularly vulnerable to SARS-CoV-2 infection, the benefits of COVID-19 vaccination seem to outweigh its risks for people with PD (17).

In our study, most patients with vaccine hesitancy were willing to receive the COVID-19 vaccination if specialists, the government, and the people around them recommended it, further supporting the notion that PD patients who are strongly against vaccines compose a tiny minority and that most patients still hold a positive attitude toward COVID-19 vaccination. To build public faith in COVID-19 vaccination, it is necessary for our leading medical organizations, such as the Chinese Medical Doctor Association (CMDA), to release an expert consensus on COVID-19 Vaccination Guidelines for patients with PD and encourage more clinical trials of Chinese COVID-19 vaccines. We believe that PD patients would be more likely to receive the COVID-19 vaccination if they can receive consistent and comprehensive information from specialists, the government, and social media.

It has been reported that the COVID-19 vaccination coverage in general population aged older than 60 years in Shanghai was much lower as compared to the nationwide (62 vs. 86%) (21). Based on these data, we speculated that patients with PD in Shanghai may similarly be at risk of vaccine hesitant. By dividing the total cohort into patients in Shanghai and patients in other cities, our study indicated that the COVID-19 vaccination rate was lower in Shanghai as expected. The multivariate model also indicated that living in Shanghai was an independent risk factor of vaccine hesitancy. The reasons underlying Shanghai's low vaccination coverage are complicated due to its broader socioeconomic status, which involves income, education and international exchanges. According to our results, the percentage of patients who trusted the safety of the COVID-19 vaccines was lower in the SH-PD group, whereas the percentage of patients who were not afraid of SARS-CoV-2 infection was markedly higher in the SH-PD group. We speculated that it might be because Shanghai is an open and modern city with diverse viewpoint and plural information channels. People may misunderstand that the COVID-19 is just a mild flu by neglecting the fact that eased restrictions in Western countries are, at least in part, based on the high vaccination coverage. This situation may be more common in Shanghai since this city is the largest economic, commercial and financial center in China with close exchange with the West. Age is another possible reason for the low vaccination rate in Shanghai. As previously mentioned, the COVID-19 vaccination coverage in China decreases with age among people older than 60 years. In our univariate model, age was a risk factor for vaccine hesitancy. Based on the latest national population census, Shanghai has the second-highest proportion of the aging population across China. Therefore, residents in Shanghai may be more inclined to refuse COVID-19 vaccination because of their older age. To reinforce public confidence in COVID-19 vaccination, local authorities need to strengthen propaganda, correct misconceptions surrounding COVID-19 and vaccines, and highlight the significance of COVID-19 vaccination for individual and community wellbeing.

Factors associated with a decrease odd of COVID-19 vaccine hesitancy included the history of flu vaccination, trust in the efficacy of vaccine, shorter PD disease duration and male gender. Similarly to COVID-19, patients with PD are at an increased risk of hospitalization for influenza. Although vaccination for common respiratory pathogens is recommended for the elderly population, vaccine hesitancy was detected in around one third of patients with PD, suggesting that there might be a general vaccination barrier among individuals with PD (17). It has been reported that compared to men, women are less likely to accept vaccination, which may be attributable to their fear of potential side events (31).

The limitations of this study include small sample size and potential sampling bias by using an online survey. This study may also have selection bias as all participants were from a single center. More large-scale, multi-center studies will be clearly needed to validate the vaccination status among patients with PD in China. In addition, clinical characteristics of PD (e.g., H-Y stage) were not assessed by physicians at the time of the online enrollment. It will be of great value to study the relationship between the severity of PD and vaccine hesitancy as well as the impact of current COVID-19 pandemic on PD progress in our future work.

Despite these limitations, our study provided the first evidence that assessed the COVID-19 vaccination coverage in PD patients and analyzed their reasons for vaccine acceptance/hesitancy. Although the COVID-19 vaccination rate was low in patients with PD, most were convinced that vaccination was beneficial and safe. Based on the available data, the benefits of COVID-19 vaccination seem to outweigh the risks for PD patients. To overcome this barrier to COVID-19 vaccination, the government and healthcare authorities need to establish public confidence in vaccines and detailed COVID-19 vaccination guidelines. On the other hand, caution should be exercised regarding potential neurological side-effects after vaccination. More clinical trials and real-life studies will be helpful to determine the safety and efficacy of COVID-19 vaccines for the PD population, which will in turn build public faith in the current vaccination campaign.

## Conclusion

Our data demonstrated that the COVID-19 vaccination coverage in patients with PD was even lower compared to the elderly population. Vaccine hesitancy was observed in around half of PD patients and interprovincial disparities in vaccine hesitation were identified. These issues will impede vaccine uptake and delay the herd immunity. In order to prevent the spread of COVID-19 and protect patients' health, great efforts are needed for health care system to enhance the public faith in vaccine and improve their willingness to vaccination.

## Data availability statement

The raw data supporting the conclusions of this article will be made available by the authors, without undue reservation.

## Ethics statement

The studies involving human participants were reviewed and approved by Institutional Review Board of Ruijin Hospital. The patients/participants provided their written informed consent to participate in this study.

## Author contributions

YW, DL, and CZ designed the study and recruited patients with PD. YZ and ZL drafted the manuscript. XW, JL, JD, and KR assisted in online survey. All authors contributed to the article and approved the submitted version.

## Funding

This study was supported by the National Natural Science Foundation of China (82171239, 81870887, 81771482, and 82101547), the Shanghai Health and Family Planning Commission Research Project (201840001), Shanghai Youth Science and Technology Talents Sailing Project (20YF1426600), Shanghai Collaborative Innovation Center for Translational Medicine (TM201904), and Innovative Research Team of High-Level Local Universities in Shanghai.

## Conflict of interest

Author KR was employed by Gyenno Science Co., LTD. The remaining authors declare that the research was conducted in the absence of any commercial or financial relationships that could be construed as a potential conflict of interest.

## Publisher's note

All claims expressed in this article are solely those of the authors and do not necessarily represent those of their affiliated organizations, or those of the publisher, the editors and the reviewers. Any product that may be evaluated in this article, or claim that may be made by its manufacturer, is not guaranteed or endorsed by the publisher.
